# Halide Site Engineering of Organic–Inorganic Hybrid Perovskites: A Facile Strategy for Frequency-Controllable Microwave Absorption

**DOI:** 10.3390/mi17050628

**Published:** 2026-05-20

**Authors:** Jinhuai Zhou, Zhi Zhang, Yao Yao, Fei Wang, Hanmin Wu, Mengjie Shi, Wenke Zhou

**Affiliations:** 1 Position Engineering Research Office, Army Engineering University of PLA, Nanjing 210007, China; 2 State Key Laboratory for Disaster Prevention & Mitigation of Explosion & Impact, Army Engineering University of PLA, Nanjing 210007, China; 3 Electromagnetic Environmental Effects Laboratory, Army Engineering University of PLA, Nanjing 210007, China

**Keywords:** organic–inorganic hybrid perovskite, electromagnetic absorption material, energy band structure

## Abstract

High-performance electromagnetic wave absorption materials are desperately needed due to the growing serious electromagnetic interference and pollution issues brought on by the quick growth of modern electronic technology and wireless communication. This work uses the organic–inorganic hybrid perovskite MAPbBr_x_I_3−x_ as a model system to address the problem of restricted loss mechanisms and the challenges in changing the absorption bandwidth of single-component wave-absorbing materials. It achieves systematic tuning of electromagnetic wave absorption performance, especially within the effective working frequency spectrum, through accurate halogen site engineering. According to the study, MAPbI_3_ (MPI), MAPbBr_1.5_I_1.5_ (MPIB), and MAPbBr_3_ (MPB), which were synthesized using the anti-solvent approach, all demonstrated exceptional microwave absorption capability, with maximum reflection loss values exceeding −37 dB, among which MPB achieves a remarkable value of −42.41 dB at 16.60 GHz. More significantly, this work shows a distinct structure-property relationship between the effective absorption peak frequency range of this series of materials and their band structure: the strongest absorption peak shows a regular blue shift as the material bandgap widens and the bromine content rises. This finding suggests that focused tailoring of the operating frequency band in wave-absorbing materials can be achieved by manipulating the band structure of perovskites by varying the halogen concentration. In addition to confirming the significant application potential of organic–inorganic hybrid perovskites in the field of microwave absorption, this study offers a novel research perspective and material template for precisely and programmably controlling the absorption frequency band of wave-absorbing materials based on their basic electronic structures.

## 1. Introduction

Micro and nanoscale dielectric loss-type microwave-absorbing materials have advanced significantly in recent years due to the rapid development of nanotechnology and composite material fabrication techniques [1,2,3]. As of right now, a number of basic investigations have shown that dielectric loss-based composite absorbers can partially meet the essential developmental requirements for electromagnetic absorbers, which include thin thickness, broad bandwidth, low weight, and strong absorption [4,5,6]. By altering the composition and proportions of the materials, the researchers can regulate the electromagnetic parameters of the composite materials, resulting in high impedance matching and attenuation loss characteristics as well as good electromagnetic wave absorption performance [7,8,9,10]. Since a large amount of research is based on the properties of composite material fabrication, the underlying electromagnetic wave absorption mechanisms within microwave-absorbing materials—such as the band structure, polarization intensity, and electrical conductivity—become significantly more complex when the materials contain a variety of different types of components. This makes it difficult to precisely determine the structure-activity relationship between a material’s intrinsic characteristics and macroscopic performance [11,12,13].

AMX_3_ (X = Cl–Br–I) is a hybrid halide perovskite material that has been identified as having a high tolerance factor. Through elemental substitution or regulated stoichiometric doping, it allows for exact control over the constituent elements of perovskite materials while preserving the basic octahedral configuration [14,15,16]. The replacement and doping of halogen elements is the main focus of X-site modification in hybrid perovskites. The bandgap of perovskite materials can be successfully adjusted by varying the composition and percentage of halide ions, as shown by an extensive study on AMX_3_ in optoelectronic engineering [17,18]. Hybrid perovskites can provide tunable photoluminescence bands in the visible light spectrum thanks to bandgap engineering accomplished by halogen substitution and doping. According to Vegard’s law, the bandgap width shows linear displacement with variations in the lattice constant [16,19,20,21].

Meanwhile, halide substitution and doping play critical roles in adjusting the crystal nucleation rate, grain size, morphology, and structural stability of perovskite. Due to the significant differences in the radii and electronegativities of different halogen ions, they will affect the supersaturation of the precursor solution and the crystal growth kinetics, thereby regulating the grain size and microstructure of the final product [22,23,24,25]. In the field of solar photovoltaics, in early single-component organic–inorganic hybrid perovskite solar cells, MAPbI_3_ exhibited the highest power conversion efficiency. However, due to its poor environmental tolerance, the tetragonal structure of MAPbI_3_ is prone to change, which leads to a decrease in its photovoltaic performance. It has been shown that doping MAPbI_3_ with halogen elements effectively improves its stability. The lattice constant of MAPbBr_x_I_3−x_ decreases when Br^−^ ions replace or partially replace I^−^ ions due to Br^−^’s smaller atomic radius than I^−^. The structure has an innate inclination to change into the cubic phase as a result of this reduction [26,27,28]. Research on the prediction and regulatory mechanism of the effective operating frequency bands of microwave-absorbing materials is still comparatively lacking in the field of electromagnetic wave absorption. Their tight relationship to the absorbing materials’ energy band structure may be verified [29,30,31]. A possible way to further clarify the fundamental connection between the physicochemical characteristics of absorbing materials and their effective absorption frequency bands is to take inspiration from the bandgap engineering via X-site modulation in hybrid perovskites. This method is crucial for precisely adjusting the working frequency ranges of microwave absorbers [32,33,34].

Thus, the anti-solvent approach was used in this study to quickly and easily synthesize MAPbBr_x_I_3−x_ (x = 0, 1.5, 3) materials with different elemental compositions. Research has shown that MAPbBr_x_I_3−x_ materials treated only by elemental doping have significant electromagnetic wave absorption performance, with a maximum absorption strength greater than −37 dB. More significantly, examination of the MAPbBr_x_I_3−x_ materials’ band structure showed that the effective absorption peak frequency shows a regular blue shift with increasing bandgap width. This discovery offers a workable method for precisely and controllably regulating the performance of electromagnetic wave absorption in such materials.

## 2. Materials and Methods

### 2.1. Materials

Lead iodide (PbI_2_, 99%), methylamine iodine (MAI, 99%), methylammonium bromide (PbBr_2_, 99%), lead bromide (MABr, 99%), gamma-butyrolactone (GBL, 99%), and anisole (C_7_H_8_O, 99.9%) were purchased from Advanced Election Technology Co., Ltd. (China, Dalian) N,N-Dimethylformamide (DMF, 99%), anisole (C_7_H_8_O, 99.9%), and gamma-butyrolactone (GBL, 99%) were purchased from Aladdin. For some compounds, additional purification procedures were not necessary.

### 2.2. Synthesis of MAPbI_x_Br_3−x_ Microcrystals

MAPbBr_x_I_3−x_ perovskite microcrystals were synthesized via the antisolvent method. By utilizing the polarity difference between the solvent and the reverse solvent, microcrystalline samples with a micron scale can be rapidly and efficiently fabricated. To prepare a 0.8 mol·L^−1^ MAPbBr_x_I_3−x_ precursor solution, the precursor powders of MAI, PbI_2_, and PbBr_2_ are first completely dissolved in a mixed solution of γ-GBL/DMF at 70 °C. The fraction of DMF in the mixed solution can be appropriately raised by increasing X. γ-GBL:DMF = 3:1 when X = 3, γ-GBL:DMF = 2:1 when X = 1.5, and γ-GBL:DMF = 1:2 when X = 0. The precursor solution was dropped into anisole at a rate of 0.5 mL/min with continuous magnetic stirring at 300 rpm at a volume ratio of 5:1 and allowed to stand for six hours. All synthesis procedures were carried out in a nitrogen-filled glove box with a temperature of 25 ± 2 °C and relative humidity < 1%. The precipitate is then repeatedly centrifugally washed with anisole following the removal of the supernatant. The product was then dried at 80 °C for 24 h to produce the required samples. MAPbI_3_ (MPI), MAPbBr_1.5_I_1.5_ (MPIB), and MAPbBr_3_ (MPB) are the usual names for the black, gray, and orange-yellow powders, respectively.

### 2.3. Structural Characterization

All as-prepared samples were characterized by multiple techniques. A Bruker D8 diffractometer (Breman, Germany) was used to perform X-ray diffraction (XRD) examination using Cu Kα radiation (λ = 1.5406 Å) at 40 kV and 40 mA, scanning from 2θ = 5° to 80°. Raman spectroscopy was used to investigate the vibrational and rotational characteristics of polar and non-polar molecules (Renishaw InVia Basis, Britain). An FLS980 Series Fluorescence Spectrometer (Edinburgh Instruments, Livingston, UK) was used to record steady-state photoluminescence (PL) spectra in the range of 300–900 nm. Electrical conductivity (EC) was measured using a TH2829C precision LCR meter (Changzhou Tonghui Electronics Co., Ltd., Changzhou, China) and an SDM-200 current density tester (Chenhua Instruments, Shanghai, China). Finally, the grain structure and micromorphology of composites and microcrystals were revealed by scanning electron microscopy (SEM, Hitachi S-4800, Hitachi (City), Japan) at magnifications ranging from 200 nm to 20 μm.

### 2.4. Microwave Absorption Characterization

Samples were uniformly mixed with paraffin wax at a mass ratio of 2:3 before being pressed into toroidal coaxial rings with an inner diameter of 3.04 mm and an outer diameter of 7 mm in order to assess electromagnetic characteristics. The coaxial ring was tested using the coaxial probe method on an Agilent PNA N5244A vector network analyzer (Agilent Technologies, Santa Rosa, CA, USA), yielding electromagnetic parameters (2–18 GHz) that included the real (ε′, μ′) and imaginary (ε‴, μ‴) components of permittivity and permeability. Reflection loss (RL) for the composites was computed using the measured values, guided by transmission line theory.

## 3. Results and Discussion

**Figure 1:**
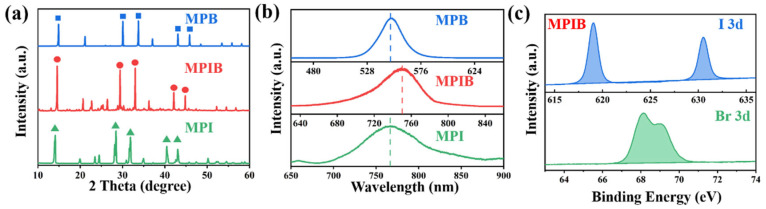
The XRD patterns (**a**), PL spectra (**b**) of MPI, MPIB, and MPB microcrystals. The characteristic XPS analysis of X-position elements in MPIB crystals (**c**).

The crystal structures and crystallinities of the MPI, MPIB, and MPB samples were characterized by XRD and PL spectroscopy, and the findings are shown in Figure 1. Figure 1a shows the XRD patterns of three crystals. It can be observed that the characteristic diffraction peaks of the three crystals are all clear and sharp. The full width at half maximum (FWHM) of the main peaks of the (110) crystal planes for MPI, MPIB, and MPB are 0.12°, 0.11°, and 0.10°, respectively. This quantitatively proves that the three crystals prepared by the reverse solvent method have high crystallinity and low intrinsic defect content. Second, the locations of the three crystals’ distinctive diffraction peaks are examined. The (110), (220), (310), (224), and (314) crystal planes of the MPI crystal are represented by the characteristic diffraction peaks at 2θ = 14.1, 28.4, 31.9, 43.1, and 43.0. It exhibits a standard tetragonal phase structure [24]. The (110), (220), (310), (224), and (314) crystal planes of the MPB crystal are represented by the characteristic diffraction peaks at 2θ = 14.8, 30.1, 33.7, 40.4, and 45.8 [35]. By observing the XRD characteristic diffraction peaks of the MPIB crystal, it can be found that since the X position is composed of I^−^ and Br^−^, all the characteristic peaks are located in the middle position between the corresponding peaks of MPI and MPB (2θ = 14.5°, 29.4°, 33.0°, 42.1°, 44.8°), which proves that MPIB is a single-phase tetragonal perovskite with uniform I/Br doping. All three crystals maintain the typical positive octahedral configuration of perovskites, and the few weak peaks not assigned in the spectra are residual trace precursors (content < 5%), which do not affect the main phase structure and properties.

Steady-state photoluminescence (PL) spectroscopy was used to characterize the band-edge emission properties of the samples. The relative change in the emission peak position can accurately characterize the trend of bandgap variation in the perovskite system [36,37]. Figure 1b displays the PL test findings for MPI, MPIB, and MPB crystals, with their distinctive emission peaks at 766.1 nm, 750.2 nm, and 550.5 nm, respectively. The correctness of the three crystals created in this section’s experiments is further validated by the fact that this finding is compatible with the reported positions of perovskite crystals in the literature [23,38]. As the Br content increases, the emission peak of PL shows a regular blue shift, clearly indicating that the band gap of the material gradually increases. Due to the larger atomic radius of I^−^ compared to Br^−^, although in the MPIB preparation process, I and Br precursors were added in a 1:1 ratio, its PL characteristic peak is closer to the MPI side. This is consistent with the nonlinear variation rule of the band gap in the double-halogen perovskite system with the composition of halogens. The XPS analysis results for three crystals are shown in Figure S1. The typical XPS peaks of both elements were examined (Figure 1c), and their peak areas were computed in order to further ascertain the ratio of I to Br in MPIB. The area ratio of these elements’ distinctive peaks was used to calculate the content ratio of these elements in MPIB crystals. The finished product’s I and Br element XPS peak areas were 0.36 and 0.39 of MPIB’s total XPS peak area, respectively, indicating a ratio of 0.48:0.52 for I^−^ to Br^−^. This is comparable to the finding that MPIB contains 0.4 mmol of PbI_2_ and PbBr_2_ precursors during preparation, suggesting that the elements I and Br are present in equal amounts in MPIB.

**Figure 2:**
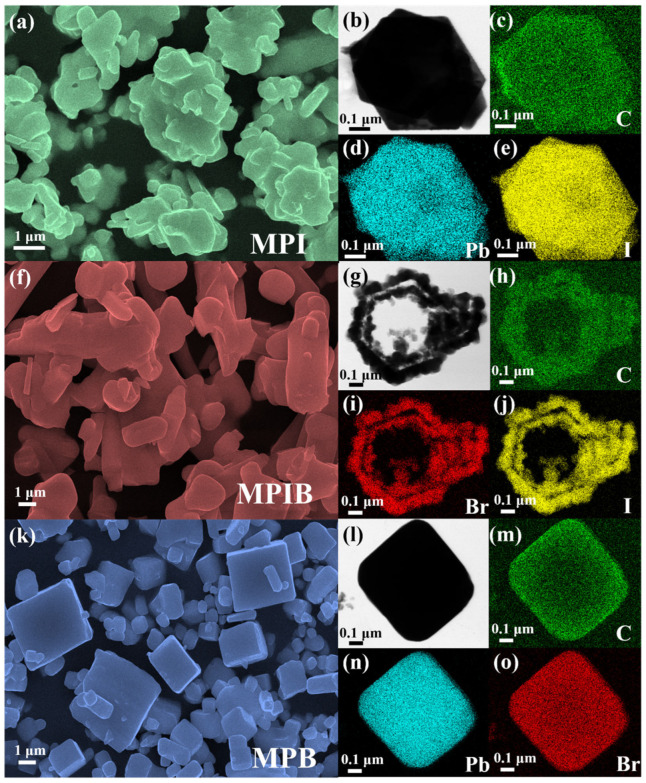
SEM images of MPI and scanned maps of element distribution (**a**–**e**); SEM images of MPIB and scanned maps of element distribution (**f**–**j**); SEM images of MPIB and scanned maps of element distribution (**k**–**o**).

We used three analytical methods to visually inspect the microstructure and elemental distribution of the three micron-sized single crystals. Figure 2 displays the test results for these crystals. Figure 2b–e,g–j,i–o show the TEM images and corresponding elemental mapping results, whereas Figure 2a,f,k display SEM images of the three crystals. Although all three types of crystals have three-dimensional block formations, their levels of regularity vary greatly. MPI microcrystals show the most irregular morphology with obvious agglomeration, while MPB microcrystals exhibit a uniform cubic block structure with negligible agglomeration. In relation to the coexistence of I and Br elements in the X position, the MPIB crystal lies halfway between the MPI and MPB crystals. Furthermore, individual microcrystals with a size of ~2 μm were selected for TEM characterization, and each crystal’s element distribution was examined. It is evident that the three crystals contain intact crystal structures and elements that are evenly dispersed. The uniform distribution of Br and I atoms in Figure 2i,j for the MPIB crystal shows that some I atoms are systematically replaced by Br atoms, creating a co-doped structure of Br^−^ and I^−^.

**Figure 3:**
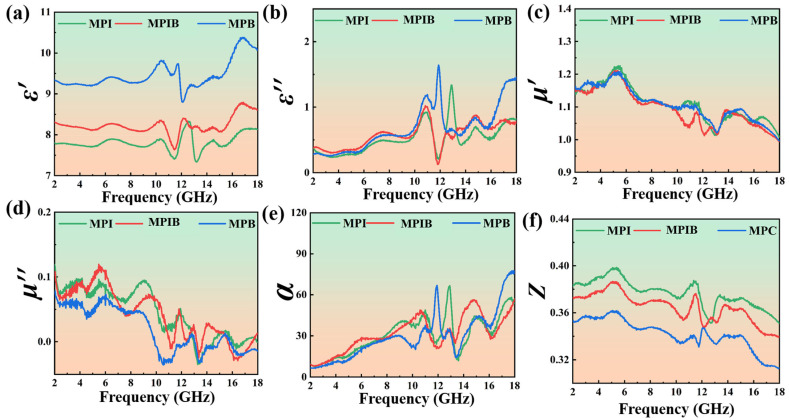
The real part of the complex permittivity ε’ (**a**); imaginary part of the complex permittivity ε’’ (**b**); real part of the complex permeability μ’ (**c**); imaginary part of the complex permeability μ’’ (**d**); attenuation loss coefficient (**e**); and intrinsic impedance ratio (**f**) of MPI, MPIB, and MPB crystals in the 2–18 GHz range.

Figure 3a–d shows the frequency-dependent complex permittivity and complex permeability of MPI, MPIB, and MPB in the 2–18 GHz range. MPB crystals show the best storage and dissipation capabilities for electromagnetic waves in the 2–18 GHz frequency band, followed by MPIB crystals, with MPI crystals performing marginally worse than the other two types of crystals. This is because the real and imaginary parts of the dielectric constant, respectively, represent the material’s capacity to store and dissipate the electric field energy of incident electromagnetic waves. Moreover, both ε′ and ε″ show negligible fluctuations in the low-frequency range (2–10 GHz) but significant variations in the high-frequency range (10–18 GHz), with fluctuation peaks being especially noticeable between 10 and 14 GHz. This suggests that the three crystals exhibit a favorable electromagnetic response to high-frequency electromagnetic waves, with the high-frequency band accounting for the majority of their electromagnetic wave absorption performance. The 10–14 GHz band is probably where the best reflection-loss performance will take place. Notably, none of the samples contain magnetic elements (Fe, Co, Ni), so their magnetic loss is negligible (μ″ < 0.1). All three crystals have low internal magnetic loss performance since their imaginary part permeability values are less than 0.1. As a result, these three materials can be examined as typical absorbing materials of the dielectric loss type. Additionally, Figure 3e,f shows the impedance matching ratios and attenuation loss coefficients of MPI, MPIB, and MPB as functions of the incident electromagnetic wave frequency. The Supporting Information has a detailed study.

**Figure 4:**
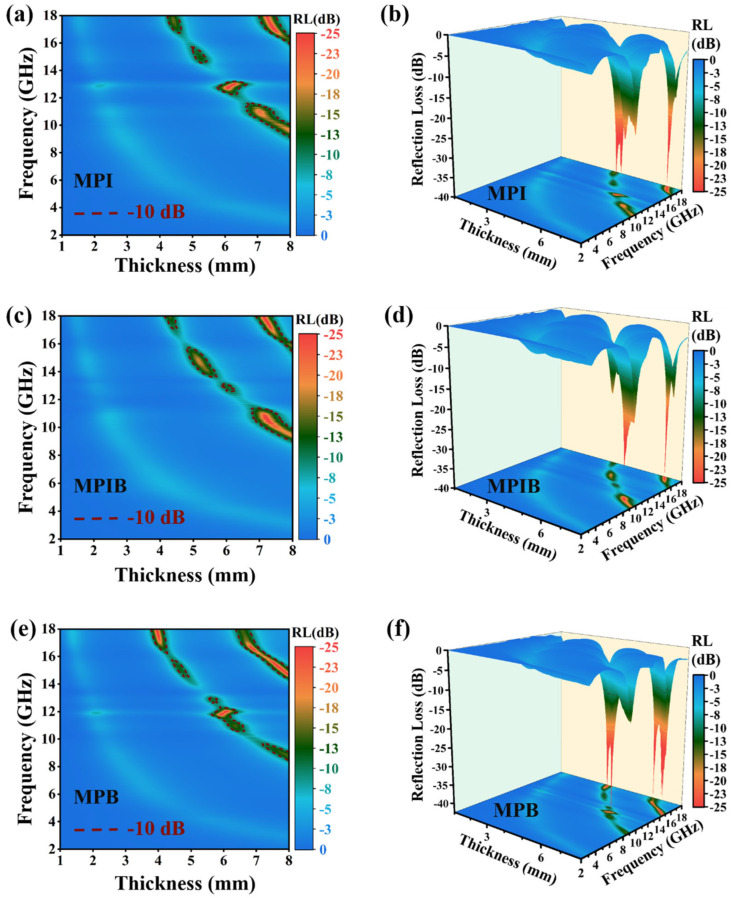
Schematic diagram of reflection loss performance of MPI crystal: 2D diagram (**a**), 3D diagram (**b**); Schematic diagram of reflection loss performance of MPIB crystal: 2D diagram (**c**), 3D diagram (**d**); Schematic diagram of reflection loss performance of MPB crystal: 2D diagram (**e**), 3D diagram (**f**).

One important factor that directly influences a material’s ability to absorb electromagnetic waves is reflection loss (RL). Stronger absorption of incident electromagnetic waves by the absorber is indicated by a lower RL value. Transmission line theory is used to determine an absorber’s RL value [39,40,41,42]:

(1) $$Z_{in}=Z_{0}\sqrt{\frac{\mu_{r}}{\varepsilon_{r}}}tanh\left(j\frac{2\pi fd}{c}\sqrt{\mu_{r}\varepsilon_{r}}\right)$$

(2) $$RL\left(dB\right)=20lg\left|\frac{Z_{in}-Z_{0}}{Z_{in}+Z_{0}}\right|$$

where *Z_in_* is the input impedance, *Z*_0_ is the characteristic impedance of free space (*Z*_0_ ≈ 377 Ω), *ε_r_ = ε*′ *− jε*′′ is the complex permittivity of the material, *μ_r_ = μ*′ *− jμ*′′ is the complex permeability of the material, *f* is the frequency of the incident electromagnetic wave, *c* is the light speed in a vacuum, and *d* is the thickness of the wave-absorbing material. The reflection loss parameters of MPI, MPIB, and MPB were computed over the 2–18 GHz frequency range in order to assess electromagnetic wave absorption capability, including maximum reflection loss and maximum effective absorption bandwidth. The principal absorption intensity and associated frequency for each material are summarized in Table 1, and the findings are shown in Figure 4. Figure 4 illustrates the high maximum reflection loss (RL_max_) values of MPI, MPIB, and MPB among single-component materials. These values are 39.83 dB at 17.04 GHz, 37.73 dB at 17.68 GHz, and 42.41 dB at 16.60 GHz, respectively. The perovskite band structure is inextricably tied to this performance. These materials have an advantage over conventional single-component absorbers because incident electromagnetic waves at these particular frequencies cause unusual vibrations and electronic transitions inside the perovskite structure, resulting in unique absorption capabilities. The three materials’ primary absorption bands are centered in the 8–18 GHz region, and they have comparatively low electromagnetic wave absorption efficiency in the 2–8 GHz band. This result is in line with findings derived from their electromagnetic parameter research. The effective absorption bandwidth (EAB, defined as the frequency range where RL ≤ −10 dB) of three single-component perovskite materials is presented in Table 2. It can be seen that the EABs of the three materials are all relatively narrow, all concentrated in the high-frequency region of 8–18 GHz, with no effective absorption detected in the 2–8 GHz band. At the thickness corresponding to their respective maximum reflection loss, MPI achieves a maximum effective absorption bandwidth of approximately 2.45 GHz; MPIB exhibits a maximum EAB of about 2.84 GHz; and MPB presents the largest maximum EAB of approximately 1.6 GHz among the three samples. No statistically significant difference in EAB was observed among the three materials. This is because of the constraints imposed by their single-component composition, which leads to weaker loss processes like conductivity loss and interfacial polarization inside the material. As a result, they are unable to effectively attenuate incident electromagnetic waves across a variety of frequency ranges due to their poor attenuation loss coefficients.

**Figure 5:**
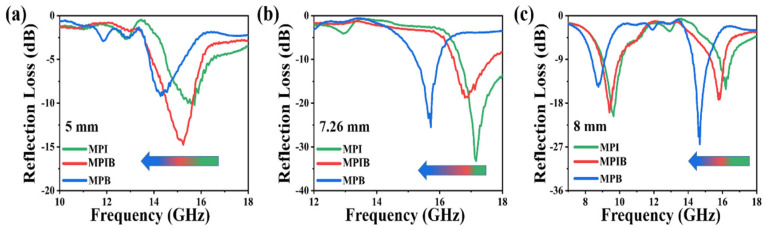
Schematic diagram of reflection loss performance of MPI, MPIB, and MPB crystals at different thicknesses: 5.00 mm (**a**); 7.26 mm (**b**); 8.00 mm (**c**).

In order to further study the influence of X-site modification of perovskite crystals on their absorption performance, the reflection loss properties of MPI, MPIB, and MPB crystals at three different thicknesses were selected. The results are shown in Figure 5. From the figure, it can be seen that under the three thickness conditions, the characteristic electromagnetic wave absorption frequency band or the strongest reflection loss peak of the MPI crystal corresponds to a frequency closer to the high-frequency range, the characteristic electromagnetic wave absorption frequency band or the strongest reflection loss peak of the MPB crystal corresponds to a relatively lower frequency, while the characteristic electromagnetic wave absorption frequency band or the strongest reflection loss peak of the MPIB crystal always lies in the middle position between the MPI crystal and the MPB crystal. Combining the characterization findings shows that the effective absorption peak frequency of MAPbBr_x_I_3−x_ shows a predictable blue shift as the bandgap width grows. Due to the fact that the different halogen elements at the X position in perovskite crystals will cause significant differences in the energy band structure of the perovskite crystals without destroying the main crystal structure of the perovskite materials, the positions of the band gap, valence band, and conduction band will change accordingly. Moreover, the different frequencies of electromagnetic waves mainly represent the different energy states of the electrons or photons they contain. Therefore, the change in the energy band structure will lead to differences in the response sensitivity of perovskite crystals to electromagnetic waves of various frequency bands. It should be noted that the relatively narrow EAB is an inherent limitation of single-component dielectric loss materials, which restricts their direct application in broadband electromagnetic protection. However, the core innovation of this work is not to pursue ultra-wideband absorption, but to establish for the first time a clear quantitative structure-property relationship between the bandgap of single-component perovskites and their microwave absorption frequency. By simply adjusting the Br^−^/I^−^ molar ratio at the X-site, the strongest absorption peak can be continuously tuned in the 10–18 GHz range without altering the basic perovskite crystal structure. This unique frequency-customizable property offers a new and facile strategy for designing absorbers for applications requiring targeted frequency absorption, such as communication anti-interference systems and electromagnetic compatibility protection for precision electronic devices.

**Figure 6:**
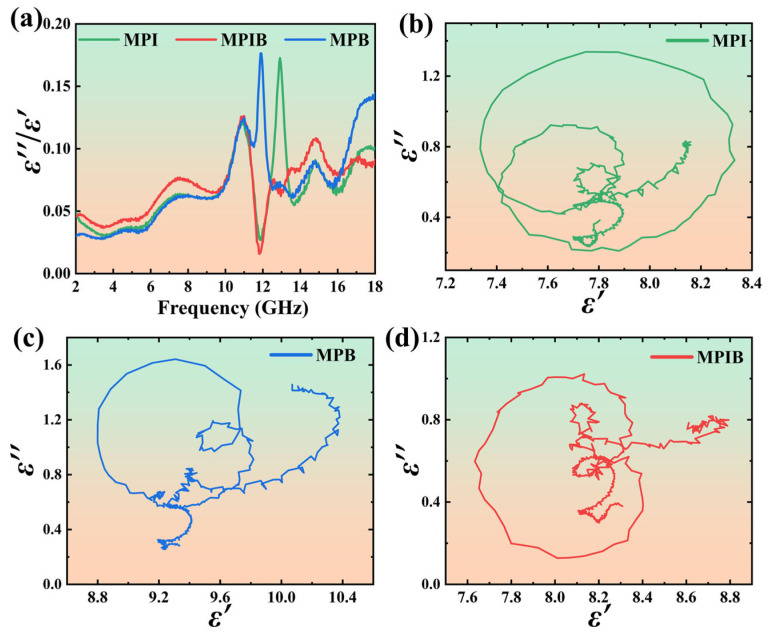
The dielectric loss tangent values of three materials (**a**); The Cole-Cole semicircles of MPI (**b**), MPIB (**c**), and MPB (**d**).

To further elucidate the microscopic origin of the dielectric loss performance of the three materials, the dielectric loss tangent values and Cole-Cole semicircles were analyzed in conjunction with Figure 6. The dielectric loss tangent (tanδ = ε′′/ε′) directly reflects the efficiency of electromagnetic wave energy conversion in dielectric loss-type absorbing materials [43,44]. The test results show that the tanδ values of the three materials in the 2–18 GHz frequency band exhibit an overall trend of “gentle rise at low frequencies, intense fluctuation at mid-high frequencies, and rebound at high frequencies”, with the overall loss level following the order MPB > MPI > MPIB, which is completely consistent with the order of their maximum reflection losses (42.41 dB, 39.83 dB, and 37.73 dB, respectively). The 10–14 GHz range is the core loss and microwave absorption band. MPB exhibits the highest loss peak (~0.18) across the entire frequency band in this interval, corresponding to its strong absorption peak of 41.27 dB at 12.0 GHz; in contrast, MPIB shows a unique loss valley feature, resulting in slightly lower absorption intensity.

The Cole-Cole semicircle model was employed to analyze the polarization relaxation behaviors of the three materials [45,46]. The ε′-ε′′complex plane curves of MPI (Figure 6b), MPIB (Figure 6c), and MPB (Figure 6d) show that all three samples exhibit three incomplete Debye relaxation semicircles with obvious high-frequency tails at the end of the curves, demonstrating that their dielectric loss originates from the synergistic effect of multiple polarization relaxations and conduction loss. The three relaxation semicircles correspond to: (1) orientational relaxation of methylammonium cations (MA^+^) within the perovskite octahedral cages; (2) dipole relaxation induced by intrinsic point defects such as halogen vacancies and lead vacancies; and (3) Maxwell-Wagner interfacial polarization relaxation at grain boundaries. In terms of relaxation strength reflected by the diameter of the outermost semicircle, MPB exhibits the strongest relaxation capability (ε′′ peak ~1.6), followed by MPI (~1.4) and MPIB (~1.1), which is fully consistent with the overall order of dielectric loss tangent values mentioned above and directly determines the difference in their maximum reflection losses. The high-frequency tails at the end of the curves for all three materials indicate that conduction loss makes a non-negligible contribution to dielectric loss. Among them, MPB shows the most prominent tail, which is attributed to the higher electronegativity and smaller ionic radius of Br^−^, which enhances carrier mobility and intrinsic electrical conductivity. The dual-halogen-doped MPIB exhibits a more irregular semicircle morphology, indicating that the coexistence of I^−^ and Br^−^ introduces additional lattice distortion and intermediate energy states in the band structure, complicating the relaxation process. This also explains the unique dielectric loss valley feature observed near 12 GHz for MPIB. The microwave absorption mechanism of the three MAPbBr_x_I_3−x_ perovskites can be attributed to the synergistic effect of polarization dissipation dominated by multiple Debye relaxations and conduction loss. The X-site halogen ion regulation achieves a precise blue shift in the relaxation characteristic frequency and absorption band by modifying the band structure, carrier properties, and defect-state distribution, further verifying the scientific validity and feasibility of bandgap engineering for frequency tuning of microwave-absorbing materials.

## 4. Conclusions

In summary, three X-site-modified MAPbBr_x_I_3−x_ perovskite materials were successfully synthesized by controlling the precursor types and ratios. MAPbBr_x_I_3−x_ demonstrates promising potential in the field of electromagnetic wave absorption, as demonstrated by the examination of its electromagnetic wave absorption characteristics. Its RL_max_ value as a single-component material was 42.41 dB at 16.60 GHz. These materials show great potential as polarization-loss media for creating high-performance electromagnetic wave absorbers by utilizing their multi-site composite features. More importantly, this work shows a special connection between the band structure of electromagnetic wave absorption materials and their absorption frequency bands, utilizing MAPbBr_x_I_3−x_ as a model system. This discovery offers important information for the creation of high-performance electromagnetic wave absorbers with quickly and accurately adjustable absorption frequencies in subsequent studies.

## Figures and Tables

**Table 1:** The main electromagnetic wave absorption intensity and its corresponding frequency of MPI, MPIB, and MPB.

Sample	Point	$\left|RLmax\right|$ (dB)	d (mm)	f (GHz)
MPI	$m_{1}$	39.83	7.49	17.04
	$m_{2}$	34.29	6.28	13.00
MPIB	$m_{1}$	37.73	7.19	17.68
	$m_{2}$	32.40	7.19	10.60
MPB	$m_{1}$	42.41	6.88	16.60
	$m_{2}$	41.27	6.07	12.00

**Table 2:** Maximum effective absorption bandwidth (EAB_max_) and its corresponding thickness of MPI, MPIB, and MPB.

Sample	d (mm)	EAB_max_ (GHz)	f
MPI	4.535	2.08	13.96–16.04
	6.425	2.45	9.44–10.12 and 11.4–11.68 & 16.51–18
MPIB	5.235	1.28	13.96–15.24
	7.37	2.84	9.72–11.16 and 16.6–18
MPB	4.15	1.6	16.4–18

## Data Availability

The data presented in this study are available in the article and Supplementary Material.

## Supplementary Materials

The following supporting information can be downloaded at: https://www.mdpi.com/article/10.3390/mi17050628/s1. Figure S1: The XPS patterns of MPI (a), MPIB (b), and MPB (c) microcrystals.
