# Supplementary material for: Halide Site Engineering of Organic–Inorganic Hybrid Perovskites: A Facile Strategy for Frequency-Controllable Microwave Absorption
Source: Micromachines (Basel). 2026 May 20;17(5):628. doi: 10.3390/mi17050628 (PMC13209366; doi:10.3390/mi17050628)
Supplement: Supplementary file 1 [file micromachines-17-00628-s001.zip › micromachines-4294135-supplementary.pdf]

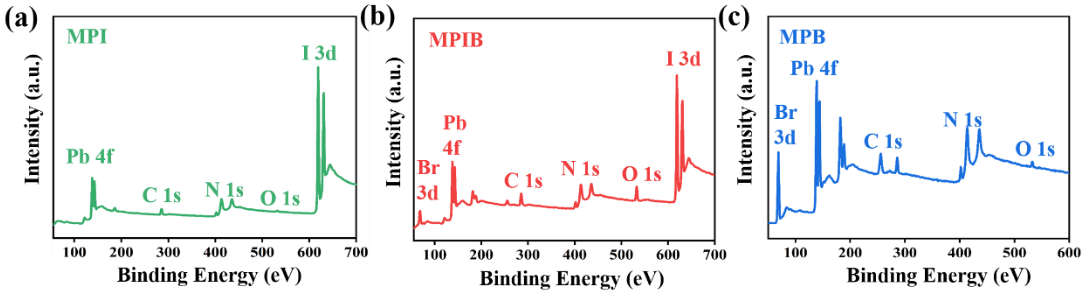

Figure S1 The XPS patterns of MPI (a)、MPIB (b)、MPB (c) microcrystals.

Figure S1 presents the results obtained from XPS analysis of the MPI, MPIB, and MPB crystals. As shown in Figure S1, the MPI crystal consists of five elements: C, N, O, Pb, and I, while the MPB crystal comprises C, N, O, Pb, and Br. The C, N, and O elements correspond to the methylamine groups occupying the perovskite A-sites. No other elements were detected in their XPS spectra.

To further investigate the electromagnetic wave absorption properties of $MAPbBr_xI_{3-x}$ perovskite materials, the attenuation constant (α) and intrinsic impedance ratio (*Z*) of MPI, MPIB, and MPB crystals were analyzed. According to transmission line theory, the relationship between α and the frequency of incident electromagnetic waves can be expressed by Equation (1):

$$\alpha=\frac{\sqrt{2}\pi f}{c}\times\sqrt{(\mu''\varepsilon''-\mu'\varepsilon')+\sqrt{(\mu''\varepsilon''-\mu'\varepsilon')^2+(\mu'\varepsilon''+\mu''\varepsilon')^2}} \tag{1}$$

where f denotes the frequency of the incident electromagnetic wave, and c represents the speed of light in vacuum. The attenuation constant is a key parameter that reflects the ability of an absorbing material to dissipate incident electromagnetic waves; a higher value indicates greater dissipation capability. *Z* can be calculated using Equations (2) and (3):

$$Z=Z_r/Z_0 \tag{2}$$

$$Z_r=Z_0\sqrt{\mu_r/\varepsilon_r} \tag{3}$$

Where Z_r represents the material's intrinsic impedance, and Z_0 denotes the free-space impedance, $\varepsilon_r=\varepsilon'-j\varepsilon''$, $\mu_r=\mu'-j\mu''$. The intrinsic impedance ratio is a key parameter that reflects the ease with which electromagnetic waves penetrate from free space into the absorbing material. Typically, when $Z>0.3$, electromagnetic waves are considered to enter the absorbing material smoothly. As shown in Figures 3e and 3f, the attenuation constants of the three crystals are relatively low, all remaining below 100 within the 2-18 GHz range. Conversely, the intrinsic impedance ratios of these materials are relatively high, exceeding 0.3 across the entire 2-18 GHz

range. This indicates that absorbers fabricated from these three materials allow substantial electromagnetic wave incidence. However, the incident electromagnetic waves cannot be effectively dissipated, allowing a significant portion to propagate through the absorber.
